# Age as a Criterion for Setting Priorities in Health Care? A Survey of the German Public View

**DOI:** 10.1371/journal.pone.0023930

**Published:** 2011-08-31

**Authors:** Adele Diederich, Jeannette Winkelhage, Norman Wirsik

**Affiliations:** 1 School of Humanities and Social Sciences, Jacobs University, Bremen, Germany; 2 Institute for Prevention Research and Social Medicine, University of Bremen, Bremen, Germany; The University of Queensland, Australia

## Abstract

Although the German health care system has budget constraints similar to many other countries worldwide, a discussion on prioritization has not gained the attention of the public yet. To probe the acceptance of priority setting in medicine, a quantitative survey representative for the German public (n = 2031) was conducted. Here we focus on the results for age, a highly disputed criterion for prioritizing medical services. This criterion was investigated using different types of questionnaire items, from abstract age-related questions to health care scenarios, and discrete choice settings, all performed within the same sample. Several explanatory variables were included to account for differences in preference; in particular, interviewee's own age but also his or her sex, socioeconomic status, and health status. There is little evidence that the German public accepts age as a criterion to prioritize health care services.

## Introduction

The patient's age is a frequently mentioned criterion when factors considered relevant for setting priorities in health care are discussed. Should elderly patients be preferred to younger patients, or younger to older ones? Is the biological age of a patient more important than the chronological age? Or should age play no role whatsoever when allocating health care resources? The present study aims at obtaining the views on these questions from a random sample of the German population. The issue is whether the public accepts age as a criterion for priority setting in health care services, and, in particular, whether differences in preference depend on the interviewee's own age, sex, socioeconomic status, or health status. This has previously been approached from both theoretical and empirical perspectives. Economic and ethical research predominantly focuses on efficiency and equity and investigates how and what type of age weight functions (e.g., single-peaked functions for relative weights; flat for equity weights) can be incorporated in the quality adjusted life year (QALY) measure used in economic evaluations of health care programs [1]–[4]. The results of most of those studies suggest that lower weight is given to older people [5]–[8].

National opinion surveys, as well as smaller surveys in local settings or among students, mainly focus on age-related preferences and attitudes and on the acceptance of age as a criterion for prioritizing patients. The results are often inconsistent and seem to depend on the design of the study and on the framing of the questions, but also on nationality [2], [8]–[10]. For instance, when asked to rank health services and treatments, higher mean priority ranks were obtained for groups involving younger patients and lower ranks for those involving older patients [11]–[13]. Similarly, when deciding between hypothetical treatment programs or between hypothetical patients, the younger were generally favored [14]–[20]. Cropper et al. [21] constructed a scenario in which patients of age between 20 and 60 years were competing for live-saving treatments. They found that interviewees wanted younger patients to be prioritized over older patients, with a peak preference for patients aged 28 years. Results from rating tasks, binary questions, and questions with several choice options are very mixed [11], [12], [16], [22]–[26]. For instance, when asked whether high-cost technology should be available to all regardless of age, Bowling [11] found that 80% of the participants agreed and 13% did not. However, 50% of the participants agreed to the statement that, if resources must be rationed, higher priority should be given to treating the young rather than the elderly; 29% disagreed. Several studies found only limited support for age based priority setting [20], [22], [23], [25], [27]–[30]. Some studies found support for preferential treatments for children and young patients [13], [31] while other studies found a strong rejection of the idea that young patients should be given priority over older patients [32]. Qualitative studies also show mixed results [33]–[37]. For instance, Kuder and Roeder [34] faced focus group members with treatment decisions between young and old patients. Participants in all groups most often chose the younger over the older patient. But, when asked for the general acceptance of age as a criterion for priority setting or for the acceptance of specific policies limiting health care based on age, the majority of the same focus group members disagreed.

While prioritizing health services has been discussed for many years in several industrial countries, in Germany it received little attention so far. In particular, German politicians and most lawyers adamantly refuse age as a criterion for allocating health care resources. Some lawyers and philosophers, however, consider age as a rather fair allocation criterion. They argue that it is fairer to ration by age than by, for instance, therapeutic benefit or severity of disease, since it affects all persons equally: everybody is getting old. Furthermore, chronological age is a transparent and objectively measurable criterion [38]. Empirical studies show that German physicians already practice age-based rationing [39], [40].

In the following, we present the views on age as a criterion for priority setting in health care of a random sample from the German population. In order to receive a broad perspective on preferences and attitudes of the German public towards age and to clarify some of the inconsistencies observed across the various studies above reported, we included questions on age that varied with respect to both complexity and form. Some questions were embedded in a health care scenario while others were rather abstract. Several explanatory variables were included to account for differences in preference, in particular interviewee's own age but also his or her sex, socioeconomic status, and health status. Moreover, discrete-choice alternatives were included in the questionnaire to measure the strength of preference. To our knowledge, the combination of a population based survey and discrete-choice questions is novel to get insight into the public's view on age as a criterion for setting priorities in health care services.

## Materials and Methods

The reported methods and results are part of a more comprehensive study on prioritizing in medicine using a multilevel iterative mixed-method design [41], [42] for combining a qualitative interview study, a quantitative survey representative for the German public and focus groups. Only parts of the middle are reported here.

### Sampling

The population survey was conducted by TNS Healthcare between July and September 2009, involving people aged 18 and over in Germany, living in private households. Data were collected with computer assisted personal interviews (CAPI). The sampling followed a three-stage random route procedure, with a design developed by ADM (Association of German Market and Social Researchers). The first stage comprises electoral wards for national elections, the second the households, and the third the individuals within the target households selected by the Kish-table method [43]. TNS Healthcare is a member of the European Society for Opinion and Market Research (ESOMAR) and is bound to its world-wide code of ethical practice, the ICC/ESOMAR International Code on Market and Social Research (http://www.esomar.org/index.php/codes-guidelines.html). In Germany, participants in social-scientific surveys give a verbal informed consent after they have been informed about the goal and content of the study, and on data protection and privacy (verbal and written). The participants' co-operation in a research project is entirely voluntary at all stages. Approval for this study was granted by the Ethics Board of the University of Bayreuth (Ethik-Kommission für Forschungsfragen der Universität Bayreuth), 95440 Bayreuth, Germany.

### Questionnaire

Thirty-four questions with 135 items were organized in the following health care and health system related themes [44]: (1) attitudes towards the German public health insurance (solidarity/shortage); (2) financial planning and insurance rates in the public health insurance; (3) areas of the health care system such as prevention, care and rehabilitation; (4) groups of persons; (5) health related behavior; (6) therapeutic benefit; (7) cost-effectiveness ratio; (8) evidence-based medicine; (9) life-threatening diseases; (10) decision-makers for allocating medical services. Both, the topics addressed in the questionnaire and the dimensions used for the discrete choice scenarios are based on results obtained from an explorative interview study with 45 members of six different stakeholder groups on prioritizing health care. Procedure and results of the qualitative interview study are found in [33], [45].

Responses were measured mainly on categorical scales (binary and multiple nominal; ordinal). A “Don't know” and “Response refused” option was offered only when the person did not respond. Unless stated otherwise these two response categories are taken together as “No answer” in the results section. In addition to questionnaire items, two different discrete-choice scenarios were presented. One scenario comprised four randomly assigned choice sets each set describing three patients and the other scenario consisted of six randomly assigned choice sets each set describing two new treatments. The sets were constructed utilizing SAS™ software.

One of the ten themes listed above was concerned with person specific characteristics as possible criteria for preferential treatment. Within this theme 30 items were constructed, including abstract questions and specific scenarios, with nine items focusing on age. The patients in the choice sets were described by six attributes, age being one of them. The following analysis focuses only on these questions and scenarios.

The whole setting was introduced by the following preface:

*We would like to know whether a specific patient or specific patient groups should receive preferential treatment if medical services are not provided for by the public health insurance to the extent they used to be.*

In case of inquiry the interviewer was informed that preferential treatment meant that this specific person will be treated first and not that the other patients are not treated at all. That is, patients might receive treatment later or with fewer resources.

A first block of questions described patients in an abstract fashion with only one person characteristic. The question “*Do you think it is justifiable to treat the following patient groups in preference to all others?*” included 18 items, three of them were concerned with age: “*elderly*”, “*children*” and “*people of working age*” nestling between the remaining items. Note that the respondents gave a yes/no answer to each of the statements, neither a ranking [11] nor a choice between mutually exclusive response options [23]. This was done to elicit preferences for a particular age group per se.

A second block of questions comprised four scenarios including age as a criterion for prioritizing a patient. Our qualitative study found age as a criterion for prioritizing patients discussed controversially. Acceptance or rejection depended greatly on the context, i.e., on the hypothetical scenarios (see Text S1 for details). Therefore, several questions in the questionnaire described the patients' situation in more detail. The first question was concerned with the order in which patients should be treated:

*Imagine two patients are life-threateningly ill, but only one treatment can be offered at the moment. What do you think: Which patient should be treated first?*

- *The younger patient*
- *The 30 years older patient*
- *A lottery decides*

“Younger” was not specified, i.e., no specific age was mentioned. The 30 years older patient's age is relative to what the participant imagined as being younger. Thus, a 40 or 50 year old patient could have easily been classified as an older patient. To find out whether respondents changed their mind to consider treatment for the older patient first when “older” was differently described, the following question was addressed only to those participants who did not give a “The younger patient” answer in the previous question:

*Assume that the older patient is* ***very*** *old. Which decision do you agree with?*

- *If the patient is* ***very*** *old, then the younger patient should be treated first.*
- *Even if the older patient is* ***very*** *old, the older patient should be treated first.*
- *Even if the older patient is* ***very*** *old, a lottery should decide.*

The next question intended to find out whether in the interviewees' view age per se might be crucial for allocating medical services, or whether additional factors, such as the general health status of the patient, should also be taken into account.

The fictive scenario and choice categories were as follows:

*Often it is reported that in England a kidney dialysis is not paid for by the national health insurance if the patient is 65 years and older, regardless of his or her general health status. Assuming that we have a similar statutory age limit in Germany, which statement would you agree with?*

- *Patients above the age limit but with a good general health status should be exempted from this regulation and the treatment should be paid for.*
- *Patients above the age limit should not be exempted from the regulation regardless of their general health status.*

There are a few areas of the medical services in Germany in which an open priority setting already exists. One example is the so-called triage-procedure applied in disaster medicine to determine treatment priorities for mass casualty incidents. The following scenario described the situation. Four different pairs of patient groups were constructed and the participants were asked whether one group should be treated in preference to the other group. The age of the patients was one critical variable (survival rate, degree of injury, and level of pain being the remaining three variables).

*Imagine the following situation: A fire broke out in an apartment house. There are many injured people, but not enough on-site medical assistance to take care of all of them at once. If you were the responsible doctor on scene, which group would you treat in preference to the other?*

*The younger casualties compared to the older casualties.*

The final scenario in this context had to do with patients in need of organ transplantation. The allocation problem becomes obvious as the number of patients waiting for an organ exceeds the number of donors. Since the set of existing criteria for allocating an organ is modified from time to time, it is interesting to know which criteria the general public would accept. The interviewees were offered the following scenario (the numbers apply for Germany):

*In 2007 about 3000 patients received a new kidney. About 8000 patients were on the waiting list, since not enough donor kidneys were available. Regarding this scarcity one might ask the question according to what criteria the organ allocation should be performed.*

Among four statements, one was concerned with the age of the patient as a criterion to allocate the organ (the remaining dealt with survival rate, waiting time, mismatch probability). The participant was asked for his/her agreement (completely agree, rather agree, rather not agree, not agree at all) to the following statement:

*Younger patients should be preferred to older patients.*

The discrete-choice sets included hypothetical patients who were characterized in terms of six factors or attributes, age being one of them: health status with levels severe and light disease, quality of life (no restrictions, restricted and severely restricted), unhealthy lifestyle (yes/no), family status (single with/out dependents (children, relatives to care for), couple with/out dependents (children, relatives to care for)), occupational status (high, medium and low), and age with levels 25, 43, 68 and 87 years. The attribute levels were combined to hypothetical patient profiles. Out of the 576 possible combinations (full factorial design) the SAS software determined a *d*-efficient design with 23 profiles. Three out of these 23 hypothetical patients (patient profiles) comprised one out of 25 choice sets ([46] for a description of the procedure and software macros). Four out of the 25 sets were randomly assigned to each questionnaire. The respondent had to indicate which patient should be treated first and who should be treated last.

The advantage of the discrete-choice method is that the participants need to trade-off several characteristics of a patient when deciding who should be treated first and thereby the relative importance of each attribute can be determined as well as the part-worth utilities for each level of an attribute [47].

Socio-demographic questions and self-reports on the respondent's life-style and health appeared at the end of the questionnaire. The self-rated health status was measured by the Short-Form Health Survey (SF-8™) [48] which consists of eight items measuring eight health domains.

## Analysis

### Socioeconomic status

The socioeconomic status was determined by the “Winkler-Index” [49]. This measure is a three-dimensional, additive, non-weighted social class index using academic/vocational education, monthly net household income and current/last occupation as indicators. Each indicator ranges from one to seven points, were one point represents the lowest and seven the highest social status; hence the Winkler-Index can take values between three and 21 points. Three social status groups with equal ranges were defined on the basis of this index: lower status (3–8 points), middle status (9–14 points) and higher status (15–21 points) [50].

### Health status

The SF-8™ Health Survey produces a physical (PCS) and a mental (MCS) component summary measure. Based on the scores each participant was categorized as above and below average, separately for each component, according to the instrument norm of 50 [51].

### Data analysis

Data analysis was carried out with SPSS™. The influence of the respondents own age on preferences was statistically tested via chi-square test for categorical data and adjusted residuals (standardized Pearson residual) as follow-up chi-square tests [52]. An adjusted residual (A-Res) that exceeds about 2 or 3 in absolute value indicates lack of fit of H_0_ in that cell [52, p. 38]. We utilized it as posthoc tests with a 5% (2≤adjusted residual<3) and 1% (adjusted residual≥3) confidence level. In addition a binary/multinomial logistic regression analysis was carried out with age, social status, sex, PCS and MCS serving as explanatory variables for the observed preferences for priority setting. For the regression analysis each explanatory variable was included in a stepwise logistic regression algorithm. Factors were sequentially removed from the model if they had a significance level above 0.05. That is, all reported main effects have a significance level of at least 5%. For better readability we omit the specific p-values but provide them for the odds ratios. The stated preference analysis in the discrete-choice sets was carried out in SAS™, resulting in part-worth utility and relative importance estimates. In a compensatory utility model, the overall utility of a hypothetical profile is the sum of the part-worth utilities of the attribute levels forming the profile. The discrete choice sets were merged for analysis, and preferences were evaluated by multinomial logit main effects models assuming independence between attributes. In order to adjust for a possible non-uniform distribution of the sets within the survey population we simulated the null model and corrected the part-worth utility estimates accordingly. Additionally we used bootstrap sampling to estimate 95% confidence intervals for the relative importance estimates.

## Results

### Sample

The number of selected addresses was 3729, of which 3% were ineligible (e.g., no private household). Of the remaining 3617 addresses, 22% of the target persons were unavailable, 13% refused to take part and 8.2% were unable for other reasons (e.g., ill), resulting in a response rate of 56.8% (2031 respondents). The sample included 1131 (55.6%) female and 900 male respondents. Mean, median and standard deviation of their age were 52, 52, and 18 years, respectively. For the analysis, respondents were grouped into three age groups: 18–29 years (14.1% of the sample), 30–59 years (46.7% of the sample) and 60 years and above (39.2% of the sample). The first group represents young adults in their early career; the second the working age group and the last the elderly. The average de facto retirement age is 60 years in Germany. A previously used finer age categorization in steps of about 10 (i.e., 18–29, 30–39, 40–49, 50–59, 60–69, 70–78, 79 and above) provided similar results. Occasionally we will come back to this classification. According to the Winkler-index, 47% of the respondents belonged to a lower social status, 39.7% to a middle social status, and 13% to a higher social status group. According to the SF-8™, 64.7% of the respondents had an above average physical health score and 81.3% an above average mental health score.

### Priority Setting

In the following, the results are presented in three parts: attitudes to age per se; attitudes towards age embedded in different scenarios; and preferences of age described as attribute in a discrete-choice setting.

### 1) Age per se

#### Elderly

**Figure 1:**
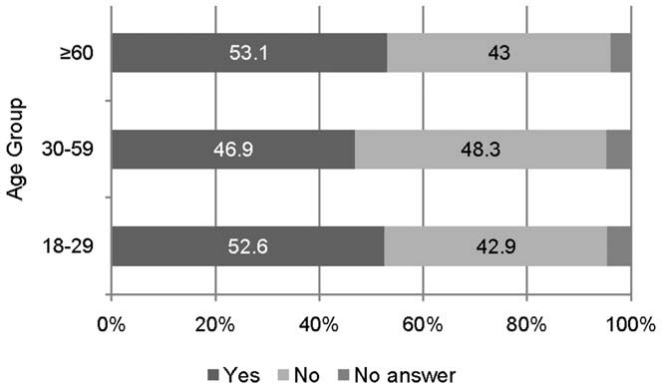
Agreement/disagreement on preferential treatment for elderly. Proportion of agreement/disagreement to elderly being preferentially treated as a function of the respondents' own age group. The majority of younger and older respondents are in favor of prioritizing elderly.

A slight majority of the population (50.2%) agreed to prefer elderly to all others, 45.4% of the population opposed it, and 4.4% of the interviewees gave no answer to the question. The response proportions as a function of the age of the participants are shown in Figure 1.

The proportion of “yes” responses is not monotonically increasing with the respondents own age but rather seems to be a U-shaped function. That is, the younger and the older participants were more likely to agree that elderly patients should receive a preferential treatment while the middle-aged participants are more likely to oppose it. However, a chi-square test revealed no dependence between age group and response category (Chi-Square(4) = 7.66, p = 0.105). A logistic regression showed main effects for socioeconomic status and physical health of the respondents. Lower and middle status participants more often agreed to prefer elderly to all others than respondents in the higher status group (odds ratio = 1.77/1.38, p = 0.000/0.027, respectively). Furthermore, respondents with a physical health score below average less often gave no informative answer than those with a PCS above average (odds ratio = 0.59, p = 0.019).

#### Children

The majority of the population (72.5%) supported the statement to prefer children to all others, 25.4% of the population objected to it, and 2.1% of the interviewees gave no informative answer to the question. The proportion of agreement is smaller for elderly (70.7%) than for younger participants (73.5%). However, no statistically significant dependence between age groups and response categories was found (Chi-Square(4) = 2.01, p = 0.733). A logistic regression revealed that sex has a significant effect on choice. Women more often preferred children to all others than men (odds ratio = 1.27, p = 0.020).

#### Persons of working-age

The majority of the population (83.6%) objected to prefer people of working-age to all others, 25.4% of the population approved it, and 2.1% of the interviewees gave no answer. Working-age people are those who finance the public health care system in Germany to a large extent, directly by their health insurance premium and indirectly by their income taxes (see Text S2 for further information about the German public health care system). However, the respondents did not consider them eligible for a preferential treatment. Bivariate analysis revealed no dependency between age group and response category (Chi-Square(4) = 4.54, p = 0.338). A logistic regression showed significant main effects for sex and physical health. Participants with a PCS score above average less often agreed to prefer working aged people to all others than those with a physical health score below average (odds ratio = 0.66, p = 0.002).

**Figure 2:**
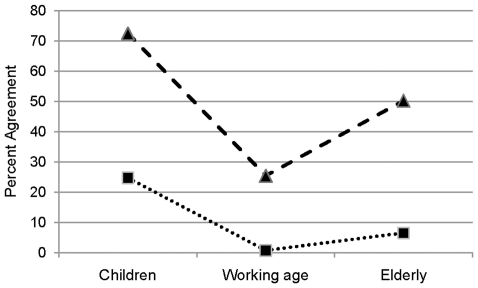
Hypothetical preference functions for age as prioritization criterion. The results are based on the proportions of agreement to treat people in different age categories preferentially. Note that the categories were not mutually exclusive. The dashed curve represents the results of total agreement observed for each statement. The dotted curve represents the proportion of agreement of those respondents who accepted preferential treatment of patients in one age category and rejected preferential treatment of patients in the two remaining age categories. They are believed to have a “true” preference for a particular age category.

Comparing the results of all three questions, some inconsistencies in interviewees' response behavior become obvious. For instance, elderly and children cannot be treated preferentially to all others at the same time. To investigate these apparent inconsistencies more closely, we divided the responders into distinct classes according to their response pattern. One group of responders may have rejected “age” as a criterion to prioritize patients categorically. In this case the respondents should have given a “no” response to all three statements. 16.9% of all respondents did so. Another group of responders may have expressed their rejection of age as a criterion by favoring all age groups. In this case we would expect a “yes” response to all three statements. 10.2% of all responders did that. Taking both results together we conclude that 27.1% of the sample rejects age per se as a criterion for prioritizing health care services. A third group may have no clear opinion on age as a prioritization criterion. Here we would expect that the respondents give a “no answer” to all three questions. This was true for only 0.1% of the respondents. For a clear opinion on a certain age group we would expect that the respondent accepts a prioritization for this group and rejects it for the two remaining age groups. Of all respondents 31.9% did that. In particular, 24.7% favored only children, 6.5% only elderly and 0.7% only persons of working age. The result of this more detailed analysis is consistent with what we have found in the pattern of the overall agreement: children receive the highest priority and people of working age the lowest. Both results are shown in Figure 2. A chi-square test revealed a dependency between response and age group (Chi-Square(4) = 17.48, p = 0.002). Participants in age group 30–59 less often agreed to give preferential treatment to elderly and more often preferred preferential treatment for children (A-Res = −3.9/3.3, respectively). Participants aged 60 years and older showed the reversed pattern: they less often agreed to give preferential treatment to children and more often preferred preferential treatment for the elderly (A-Res = −2.9/2.9, respectively).

There are more response patterns possible, however, of less importance here, in particular those including a “no answer” for one or two age groups.

If we assume that the amount of agreement with any of the three questions somehow reflects the respondents' preference strength for specific age categories when setting priorities in health care services, then the function shown in Figure 2 deviates from what is proposed in the literature. It is neither flat nor decreasing as a function of age, nor single-peaked with the peak somewhere in the middle as described, for instance, by Murray [53], Williams [1], or Tsuchiya et al. [4]. Note, however, that those studies required a comparison and ranking of age groups from the interviewees. We return to this issue when we discuss the results of the discrete-choice task.

### Age embedded in scenarios

#### Scenario 1: Life-threatening illness

**Figure 3:**
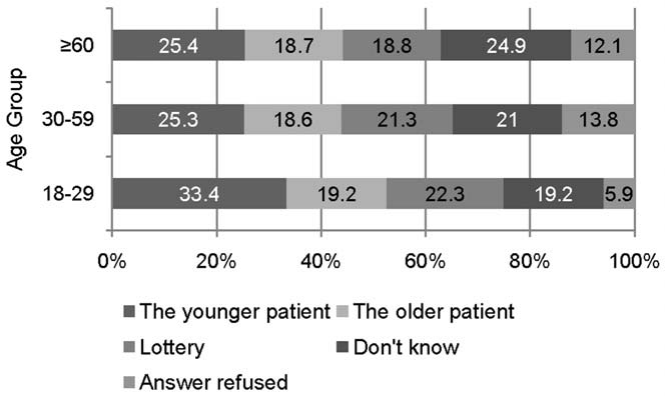
Agreement to statements for preferential treatments in the life-threatening illness scenario by respondents' age.

The results to the life-threatening illness scenario asking for preferential treatment are as follows: 26.5% of the sample considered “The younger patient” first, 18.7% “The 30 years older patient”, and 20.5% of the participants preferred “A lottery decides”. 22.3% opted for the “Don't know” response and 12.1% refused to answer this question. A chi-square test revealed a dependence between age group and response category (Chi-Square(8) = 23.74, p = 0.003). The response proportions as a function of age groups are presented in Figure 3.

The adjusted residuals show that significantly more participants in age group 18–29 preferred younger patients to be treated first (A-Res = 2.9). Furthermore, participants in this age group less often refused to give an answer (A-Res = −3.4), and participants in age group 30–59 more often refused to answer this question (A-Res = 2.3). Respondents of age 60 and above gave more often a “Don't know” response (A-Res = 2.3). A logistic regression revealed main effects for the socioeconomic status and the age of the participants. Respondents with lower and middle status more often preferred older patients to be treated first than respondents in the higher status group (odds ratio 3.29/2.45, p = 0.000/0.001, respectively). Respondents in age group 18–29 gave less often a “Don't know” answer and less often refused the answer than the remaining age groups (odds ratio = 0.59/0.37, p = 0.007/0.001, respectively).

The following question described “older” differently and was addressed to those participants who did not opt for “The younger patient” in the previous question; thus, the sample size is n = 1493, 73.5% of the original sample. Now 17.3% of the participants preferred the younger patient, 21.7% still wanted the older patient to be treated first, 27.1% opted for the lottery, 21.2% gave a “Don't know” response and 12.7% refused to answer the question. No dependency between age groups and response categories could be observed (Chi-Square(8) = 11.58, p = 0.171).

Comparing the response pattern for both questions gives the following result: 37.6% of the respondents gave a similar answer for both questions (30.8% participants gave a “no response”; 2.3% preferred the older patient over the younger and 4.5% decided for the lottery). 17.1% of those who decided for the 30 years older patient in the first question preferred the lottery in the second, and about the same number of participants (17.7%) reversed their preferences for the chance option to the older patient. 4.4% of the interviewees who decided for the 30 years older patient preferred the younger patient in the second framing; similarly, 4.2% of those who previously preferred the chance option now decided for the younger patient. 8.7% of those who gave a “no response” preferred the younger patient when the older patient was described as very old. The details are shown in Table 1. No dependency between the participants' age group and the response pattern could be observed (Chi-Square(38) = 38.80, p = 0.433).

**Table 1:** Proportion of agreement to statements for preferential treatments in the life-threatening illness scenario for two different questions.

	Question asked first		
Question asked second	30 years older patient	Lottery	No response
Younger patient	4.4	4.1	8.7
Very old patient	**2.3**	17.7	1.7
Lottery	17.1	**4.5**	5.4
No response	1.6	1.5	**30.8**

Bold numbers indicate the proportion of respondents who gave similar answers in both situations.

Two points are worth mentioning. First, the proportion of “no answer” is relatively high, in particular compared to the proportions obtained for the abstract questions. For those questions a specific age group was compared to “all others”, i.e., a not further specified population rather than two patients described in a presumably concrete context. Second, the proportion of those favoring a lottery for medical treatments seems relatively high compared to other studies [28], [30].

The previous questions aimed at finding out whether the relative age difference or the absolute age of a person is crucial for a decision. To determine how “very old” was interpreted a follow-up question asked the participants from what age on they would consider a patient as “very old”.

Of all respondents 95.5% indicated a specific age. The mean of it was 82.5 years with a standard deviation of 8.6 years and a median and mode of 80 years; values ranged from 50 to 110 years. Cumulated relative frequencies are shown in Table 2.

**Table 2:** Cumulated relative frequencies for reported years to be labeled as “very old”.

**Years**	≤60	65	70	75	80	85	90	95
**Cumulated %**	2.5	4.0	12.5	20.7	52.4	67.8	92.2	95.0

Only 2.5% of the participants called a person 60 years and younger as very old. 31.7% of the interviewees considered a person between 75 and 80 as “very old” and for 5% of the participants a person needed to be 95 and older to be “very old”. Although the mean of the reported age slightly increased (79, 80, 82, 83, 84, 85, 85) with increasing age of the participants (18–29, 30–39, 40–49, 50–59, 60–69, 70–78, 79 and above) the range, from 50 to 110 years, was about the same for all age groups (the standard deviation is about 8 years in each group).

#### Scenario 2: Fixed age limit

**Figure 4:**
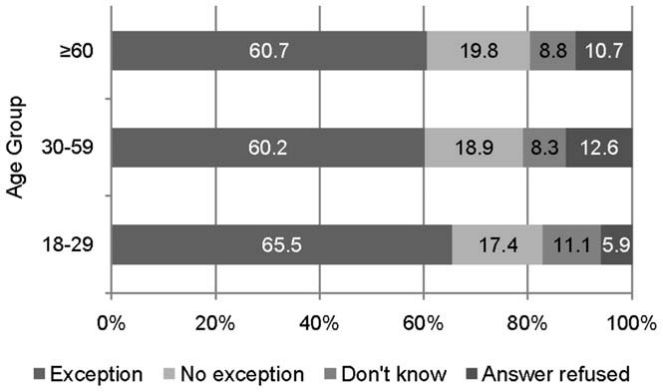
Agreement/disagreement allowing an exemption of fixed age limit for health services by respondents' age.

The results to the second scenario - asking for an exemption of a fixed age limit for patients with a good general health status for providing health services - are as follows: 61.2% of the participants agreed with the exemption. The proportion of those who disagreed, i.e., who would deny the patient above 65 years a paid treatment even with a possibly good health status seems relatively high (19.1%). Furthermore, the proportion of non-responders also seems relatively high (19.7%). A chi-square test revealed a statistically significant dependence between age group and response category (Chi-Square(6) = 12.66, p = 0.049). The response proportions as a function of the participants' own age group are shown in Figure 4.

Interestingly, the largest proportion of supporters for an exemption was found in the youngest age group 18–29 (65.5%) and the lowest proportion of supporters in the oldest age group 79 and above (53.5%). Here we chose the finer age group classification to show the difference in acceptance for the youngest and oldest interviewees. However, the adjusted residuals showed no difference between age groups with respect to acceptance and rejection of an exemption. Significant differences were only found for refusing the response. Less participants in age group 18–29 refused to give a response to this question (A-Res = −2.9) and more participants in group 30–59 refused to give a response (A-Res = 2.3). A logistic regression revealed significant main effects for the mental health score, the socioeconomic status, and the age of the participants. Participants with a MCS score above average less often agreed with an exemption and gave less often a “Don't know” answer than respondents with a MCS score below average (odds ratio = 0.68/0.57, p = 0.019/0.017, respectively). Participants with a higher socioeconomic status less often refused to answer than participants with a lower and middle status (odds ratio = 0.46/0.59, p = 0.003/0.032).

#### Scenario 3: Triage

**Figure 5:**
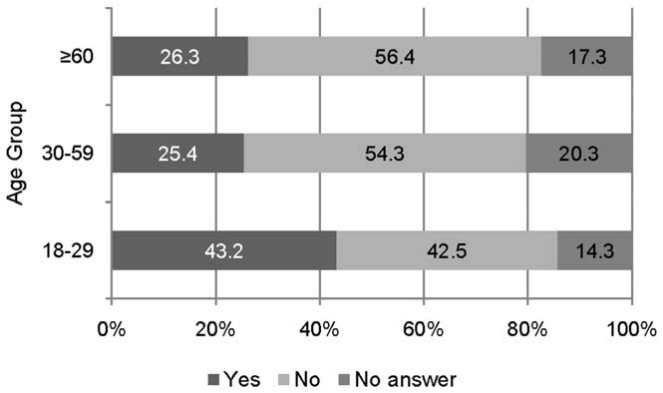
Agreement/disagreement of preferential treatment for younger casualties by respondents' age.

In the triage situation, 28.3% of the participant would treat the younger casualties first. The majority (53.5%) did not agree with this statement and 18.3% did not provide information to it. Age groups and response categories are statistically dependent (Chi-Square(4) = 39.29, p<0.000). Response proportions as a function of age groups is presented in Figure 5.

The adjusted residuals showed that respondents in age group 18–29 would treat the younger casualties in preference to the older casualties more often than the remaining age groups (A-Res = 6.1); they also showed the lowest proportion of rejecting this statement (A-Res = −4). Respondents in age group 30–59 more often gave a not informative response than the other age groups (A-Res = 2.2).

A logistic regression showed significant main effects for age group and socioeconomic status. Participants in age group 18–29 more often agreed to prefer younger casualties than the participants in the two other age groups (odds ratio 2.26, p<0.000). Participants with a lower socioeconomic status less often gave a not informative answer than participants with a middle and higher socioeconomic status (odds ratio 0.615, p = 0.009).

#### Scenario 4: Organ transplantation

**Figure 6:**
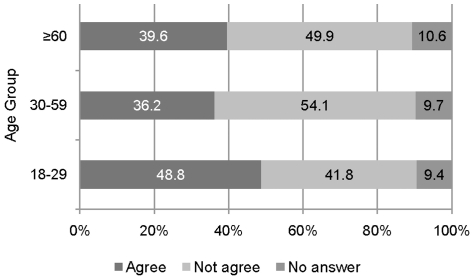
Agreement/disagreement of preferential treatment of younger patients waiting for donor kidney by respondents' age.

Of all participants 50.7% disagreed with the statement that younger patients should be preferred to older patients in the allocation of donor kidneys (26.3% rather not agreed, 24.4% not agreed at all); 39.3% agreed to it (15.9% completely agreed, 23.4% rather agreed). The remaining 10% did not give an informative answer. Figure 6 shows the response proportions as a function of age groups.

A chi-square test revealed a dependence between age group and response category (Chi-Square(4) = 16.11, p = 0.003). The adjusted residuals showed that significantly more participants in age group 18–29 preferred younger patients than the independence hypothesis predicts (A-Res = 3.6). The same age group less often disagreed with the statement (A-Res = −3.3). Respondents of age group 30–59 less often agreed (A-Res = −2.7) and more often disagreed (A-Res = 2.9) with the statement, that younger patients are preferentially treated. A logistic regression revealed significant main effects for age group and mental health scores. Participants in age group 18–29 more often agreed with the statement that younger patients should be preferred to older patients than the other two age groups (odds ratio 1.56, p = 0.002). Respondents with a MCS score above average less often agreed with the statement than respondents with a MSC score below average (odds ratio 0.718, p = 0.006).

The results show that age, as a criterion for prioritizing patients described in various medical scenarios, is not accepted by the majority. Statistical tests showed that the respondents' own age in the youngest age group (18–29) influenced the response. They tend to favor the younger patient. However, the explanatory variables *socioeconomic status* and *health status* seems to be better predictors for differences in preference than the person's own age.

### Age as a factor in discrete-choice sets

**Figure 7:**
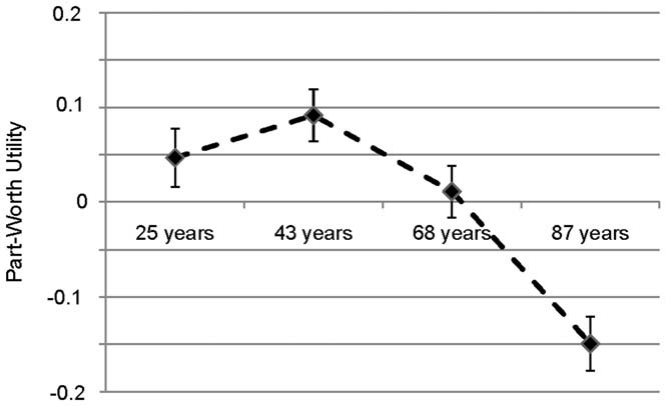
Part-worth utilities and their 95% confidence interval for the factor *age*.

The final part analyzes age as a prioritization criterion in the context of discrete-choice sets, whereby we focus on the results for age only. On a normalized scale from 0 to 100, age received a relative importance weight of 12.03 (for comparison, “health status” received the highest weight, 49.97, followed by “quality of life”, 24.65; “family status”, 7.92, “occupational status”, 4.60 and “lifestyle”, 0.82. Details are found in Table S1). The part-worth utilities of the four age values are presented in Figure 7 (statistical details are found in Table S2). They provide a quantitative measure of the preference for each attribute level, with larger values corresponding to greater preference.

When varying the age levels and keeping the levels of the remaining attributes fixed, a 43 year old patient should be treated first, a 25 year old patient second, a 68 year old patient third, and an 85 year old patient should be treated last. That is, the most preferred age here was 43 years – people of working age. This group, however, is the group least preferred when the respondents answered abstract age-related questions (see above). Although the age categories in the discrete-choice scenario are not exactly the same as in Figure 2, it becomes obvious that the preferences for age groups are reversed. No significant differences between the respondents' age groups for the part-worth utility estimates were observed. However, the point estimates were the highest when the hypothetical patient's age group corresponded to the respondents' age group.

## Discussion

Prioritizing health care services has been discussed for many years in several countries. Although the German health care system has budget constraints similar to many other countries worldwide, a discussion on prioritization has not gained the attention of the public yet. The present study may be a first step towards this goal: a population survey, part of a comprehensive multilevel iterative mixed-method design, which combines qualitative and quantitative methods, aiming at eliciting attitudes and preferences towards proposed criteria for prioritizing health services. A qualitative interview study explored in detail the considerations underlying priority setting decisions in health care of different interest groups, and found that age was discussed most controversially, and acceptance and rejection depended greatly on the context. This criterion was investigated here in detail by using different types of questionnaire items, from abstract age-related questions to health care scenarios and discrete choice settings, all performed with the same sample. Since the sample is representative, attitudes towards age as a criterion for setting priorities in health care as well as differences between various groups of people can now be generalized for the German population.

When asked whether patients of a specific age group should receive preferential treatment to all others, the highest proportion of agreement was observed for children, the lowest for people of working age, and those for the elderly somewhere in between. Since these three age groups were not mutually exclusive, it turned out that the vast majority agreed to prioritize children to all others but, at the same time, a slight majority also agreed to prioritize elderly to all others. While the first result is not surprising, the latter is in the first instance. The results of the other studies reported above found that, if a certain age (group) was accepted for prioritizing health care services, then it was for the younger patients, never for the elderly. However, a more detailed analysis revealed that only a minority had a clear preference for a specific age group. Still, the agreement was at its lowest level for treating people of working age preferentially.

The results on the four health service scenarios showed little evidence that age may serve as criterion for prioritizing health services in Germany. In fact, there was no majority supporting age as prioritization criterion in any of the scenarios. The proportion of “no answer” (i.e., “Don't Know” and “Answer refused”) was relatively high in most of the scenarios, up to about 31% in the Life-Threatening Illness scenario. For comparison the proportion of a “no answer” for the abstract questions was between 2% and 4%. This may be interpreted as a way out for the interviewees when a decision had to be made between two patients, one described as young and one as old, rather than between two anonymous subpopulations, one described as an age group and one as “all others”.

The Life-Threatening Illness scenario where only one treatment could be offered at the moment, not only had the highest proportion of “no answer” but the proportion of those favoring a lottery for medical treatments seemed relatively high compared to related studies [28], [30]. Again, this may be interpreted as kind of avoidance behavior since a decision for one person means a decision against the other. Nord et al. [23] offered three options in their life threatening scenario: the younger, against/for the older and equal priority. Overall, the equal priority option received the largest acceptance, its strength depending on the concrete context. In the Life-Threatening Illness context we also investigated what “older” and “old age” meant to the interviewees. To call a person “very old” the mean (median) was 82.5 (80) years; the estimates did not depend on the persons own age group. It seems relatively high but reflects the life expectancy in Germany which is about 80 for men and woman taken together.

The Fixed-Age scenario asked for acceptance or rejection of an exemption for treating people with general good health beyond a certain age. Although the majority favored an exemption, about 20% of the respondents opt for no exemptions. Obviously, such a strict rule is not even applied in countries where a similar regulation exists for many years. The Triage scenario showed generally no evidence for accepting age as a prioritization criterion. However, the observed proportion supporting a preferential treatment for younger casualties is far higher for the respondents in age group 18–29 than for the remaining age groups. Indeed, the difference in proportion for accepting younger age for preferential treatment as a function of the interviewees' own age is highest for this scenario.

Ratcliffe [16] found that 66% of participants agreed that younger people should be prioritized over older ones in organ allocation whereas our Organ Transplantation scenario showed no evidence for accepting age as a prioritization criterion.

We expected the respondents' own age group to be the most critical explanatory variable for response behavior and hypothesized that a person of a certain age group prefers his or her own age [11], [25]. While our results points in that direction for age group 18–29, it could not be observed for the remaining age groups, in particular not for people of working age. Socioeconomic status explained some of the variance, but not for all questions considered here. The same holds for the explanatory variable health status. Sex differences could not account for variability except for one question: preferential treatment for children.

Finally, the discrete-choice sets enabled us to estimate the importance weight for the factor “age” and the preference strength of its levels. A major advantage of this approach is that participants consider several attributes jointly, compare them, and make trade-offs to reach a decision. Opting for preferential treatment of the elderly over all others and of children over all others at the same time, as it was done for the abstract questions, is logically impossible. Furthermore, specific prioritization criteria are less obvious, like for the abstract questions and the health care scenarios. When asking participants directly, social desirability may affect the response (e.g., children yes, but people of working-age not). On a scale from 0 to 100, age received a relative weight of about 12, while “health status” and “quality of life” obtained weights of about 25 and 50. On the other hand, age is more important than the remaining three attributes. Indeed, it is as important as taking social responsibility (family status), socioeconomic status, and the patient's own responsibility for the illness together. Schwappach [17] found related results in a conjoint-analysis like approach with budget allocation, conducted with 150 students from medical and economic faculties. Age received the lowest relative weight (9) while quality of life after treatment received the highest weight of about 33 (the remaining attributes with relative important weight were healthy lifestyle (25); socioeconomic status (23) ; life expectancy after treatment (13); prior receiver of costly treatments (16)). Age has some weight but there are other, more important criteria when allocating health care resources.

The part-worth function (Figure 7) resembles in shape the so-called productivity ageism function, proposed by Tsuchiya et al. [4] which gives priority to young adults because they are more productive. The ordinate units are relative values of life-years rather than utilities for age values. The productivity ageism function in turn is related to the age weight function proposed by Murray and Lopez [3] to weigh DALYs (disability adjusted life years). The rationale of the so called efficiency-based age weighting functions is that health gain at different ages is valued differently according to the expected level of productivity at each age. Productivity is defined in a broad sense including home and society [4, p. 688]. This, however, is in stark contrast to the response pattern observed here for the age per se questions where the most productive group received the lowest rates of agreement. We cannot settle this issue from the survey data. Therefore, in our multilevel iterative mixed-method design the next stage is to include focus groups to gain insight into the underlying arguments for apparently inconsistent reasoning. To conclude: Although the agreement to prioritize a specific age group or an individual patient of a certain age varied slightly with the context in which age was embedded, there is little evidence that the German public accepts age as a criterion to prioritize health care services.

## Supporting Information

Table S1 Relative importance of the six attributes describing the patients' profile including the 95% confidence intervals.
(DOC)

Table S2 Part-worth utilities for the attribute age including the 95% confidence interval.
(DOC)

Text S1 Qualitative study.
(DOC)

Text S2 German public health care.
(DOC)
