# Supplementary material for: Age as a Criterion for Setting Priorities in Health Care? A Survey of the German Public View
Source: PLoS One. 2011 Aug 31;6(8):e23930. doi: 10.1371/journal.pone.0023930 (PMC3164130; doi:10.1371/journal.pone.0023930)
Supplement: Table S1 — Relative importance of the six attributes describing the patients' profile including the 95% confidence intervals. (DOC) [file pone.0023930.s001.doc]

Table S1: Relative Importance of the Six Attributes Describing the Patients’ Profile Including the 95% Confidence Interval

| **Attribute** | **Relative Importance (in %)** | **Lower Limit of 95% Confidence Interval** | **Upper Limit of 95% Confidence Interval** |
| --- | --- | --- | --- |
| Age | 12.03 | 10.05 | 13.99 |
| Unhealthy Life Style | 0.82 | 0.04 | 2.28 |
| Family status | 7.92 | 5.99 | 9.76 |
| Quality of Life | 24.65 | 22.89 | 26.26 |
| Severity of Illness | 49.97 | 47.74 | 52.00 |
| Occupational Status | 4.60 | 2.91 | 6.20 |
