# Supplementary material for: Age as a Criterion for Setting Priorities in Health Care? A Survey of the German Public View
Source: PLoS One. 2011 Aug 31;6(8):e23930. doi: 10.1371/journal.pone.0023930 (PMC3164130; doi:10.1371/journal.pone.0023930)
Supplement: Table S2 — Part-worth utilities for the attribute age including the 95% confidence interval. (DOC) [file pone.0023930.s002.doc]

Table S2: Part-Worth Utilities for the Attribute Age Including the 95% Confidence Interval

| **Attribute Level** | **Part Worth Utility** | **Lower Limit of 95% Confidence Interval** | **Upper Limit of 95% Confidence Interval** |
| --- | --- | --- | --- |
| 25 years | 0.052 | 0.021 | 0.083 |
| 43 years | 0.086 | 0.058 | 0.113 |
| 68 years | 0.009 | -0.018 | 0.036 |
| 87 years | -0.147 | -0.176 | -0.118 |
