# Supplementary material for: Age as a Criterion for Setting Priorities in Health Care? A Survey of the German Public View
Source: PLoS One. 2011 Aug 31;6(8):e23930. doi: 10.1371/journal.pone.0023930 (PMC3164130; doi:10.1371/journal.pone.0023930)
Supplement: Text S1 — Qualitative study. (DOC) [file pone.0023930.s003.doc]

Using semi-structured interviews, Heil et al. [33] asked participants whether any specific groups should be prioritized or posteriorized when allocating health care resources. Twenty-four out of 45 participants brought up the patient’s age as possible criterion. Sixteen participants wanted to prioritize young people and children while twelve participants wanted to prioritize older people. Five participants favored young and older patients at the same time. Seven participants mentioned to posteriorize elderly. When asked to decide between two hypothetical patients, described in terms of age and severity of health, and applying for a rehabilitative intervention, the majority favored the 58 years old patient who suffers from backache and asthma over the 41 years old patient who suffers only from backache (21 versus 17 participants). Only two out of 21 participants voted for the older patient because of her old age, three out of 21 mentioned age to posteriorize her; eight out of 17 participants who preferred the younger patient did so because of her young age. However, the decisive factor that led to the preferential treatment of the former patient was not her age but the severity of her disease: 17 out of 21 participants preferred the older patient because of her comorbidity.
