# Supplementary material for: Age as a Criterion for Setting Priorities in Health Care? A Survey of the German Public View
Source: PLoS One. 2011 Aug 31;6(8):e23930. doi: 10.1371/journal.pone.0023930 (PMC3164130; doi:10.1371/journal.pone.0023930)
Supplement: Text S2 — German public health care. (DOC) [file pone.0023930.s004.doc]

In Germany, nearly 90% of the population is covered by the statutory health insurance (about 10% are privately insured). The amount of insurance contributions mainly depends on the gross income of the insured person and is co-financed by employer and employee. The claim for benefits is independent of the amount of insurance contributions. Children in the statutory health insurance are basically exempt from paying a premium and are covered by the so called family coinsurance. Retired people used to be exempt from paying a health care premium but were fully covered. Since 1983 (‘21. Rentenanpassungsgesetz’), they pay a small health insurance premium.
